# Spatiotemporal maintenance of flora in the Himalaya biodiversity hotspot: Current knowledge and future perspectives

**DOI:** 10.1002/ece3.7906

**Published:** 2021-07-17

**Authors:** Moses C. Wambulwa, Richard Milne, Zeng‐Yuan Wu, Robert A. Spicer, Jim Provan, Ya‐Huang Luo, Guang‐Fu Zhu, Wan‐Ting Wang, Hong Wang, Lian‐Ming Gao, De‐Zhu Li, Jie Liu

**Affiliations:** ^1^ CAS Key Laboratory for Plant Diversity and Biogeography of East Asia Kunming Institute of Botany Chinese Academy of Sciences Kunming China; ^2^ Germplasm Bank of Wild Species Kunming Institute of Botany Chinese Academy of Sciences Kunming China; ^3^ Department of Life Sciences School of Pure and Applied Sciences South Eastern Kenya University Kitui Kenya; ^4^ Institute of Molecular Plant Sciences School of Biological Sciences University of Edinburgh Edinburgh UK; ^5^ CAS Key Laboratory of Tropical Forest Ecology Xishuangbanna Tropical Botanical Garden Chinese Academy of Sciences Xishuangbanna China; ^6^ School of Environment, Earth and Ecosystem Sciences The Open University Milton Keynes UK; ^7^ Institute of Biological, Environmental and Rural Sciences Aberystwyth University Aberystwyth UK; ^8^ University of the Chinese Academy of Sciences Beijing China; ^9^ Kunming College of Life Sciences University of Chinese Academy of Sciences Kunming China

**Keywords:** biodiversity hotspot, climate change, elevational gradient, Himalayan flora, mountain ecosystem, spatiotemporal diversification

## Abstract

Mountain ecosystems support a significant one‐third of all terrestrial biodiversity, but our understanding of the spatiotemporal maintenance of this high biodiversity remains poor, or at best controversial. The Himalaya hosts a complex mountain ecosystem with high topographic and climatic heterogeneity and harbors one of the world's richest floras. The high species endemism, together with increasing anthropogenic threats, has qualified the Himalaya as one of the most significant global biodiversity hotspots. The topographic and climatic complexity of the Himalaya makes it an ideal natural laboratory for studying the mechanisms of floral exchange, diversification, and spatiotemporal distributions. Here, we review literature pertaining to the Himalaya in order to generate a concise synthesis of the origin, distribution, and climate change responses of the Himalayan flora. We found that the Himalaya supports a rich biodiversity and that the Hengduan Mountains supplied the majority of the Himalayan floral elements, which subsequently diversified from the late Miocene onward, to create today's relatively high endemicity in the Himalaya. Further, we uncover links between this Miocene diversification and the joint effect of geological and climatic upheavals in the Himalaya. There is marked variance regarding species dispersal, elevational gradients, and impact of climate change among plant species in the Himalaya, and our review highlights some of the general trends and recent advances on these aspects. Finally, we provide some recommendations for conservation planning and future research. Our work could be useful in guiding future research in this important ecosystem and will also provide new insights into the maintenance mechanisms underpinning other mountain systems.

## Graphical abstract

Although the Himalaya harbors one of the world's richest floras, our understanding of the origin and spatiotemporal maintenance of this high biodiversity remains poor. This review provides a concise synthesis of the origin, distribution, and climate change responses of the Himalayan flora. Additionally, we suggest some conservation interventions that would be useful for ecosystem management and future research.

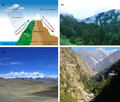

## 1 INTRODUCTION

Mountain ecosystems are important not only as biodiversity reservoirs (Myers et al., 2000), but also as sources of raw materials and for their associated sociocultural value (Stepp et al., 2005). Mountain building (orogeny), a process that is driven by plate tectonics, creates climatic, landscape, and ecological gradients that are fundamental for species evolution (Spicer, 2017). Owing to this topographic and environmental complexity, mountains support a significant one‐third of all terrestrial biodiversity (Spehn et al., 2011). For instance, where the elevational gradient is strong, drastic altitudinal climate variation over very short distances may create different environmental niches very close together, although such juxtapositions can also result from simple geometric constraints on species distribution boundaries without the action of any environmental gradients (Brehm et al., 2007; Colwell et al., 2004), potentially driving divergence and speciation (Doebeli & Dieckmann, 2003; Funk et al., 2016). Major mountain ranges, including the Himalaya, Andes, and Alps among others, are all known to be biodiversity hotspots (Mittermeier et al., 2004; Myers et al., 2000). The term “biodiversity hotspot” refers to an area with high concentrations of endemic species that face a significant risk of habitat loss (Myers, 1988; Myers et al., 2000; but see Marchese, 2015 for a more inclusive definition). The focus of the current review is the Himalaya, which harbors one of the world's richest montane floras, in addition to supporting the livelihoods of more than 1 billion people inhabiting the adjacent lowlands (Xu et al., 2009).

**FIGURE 1:**
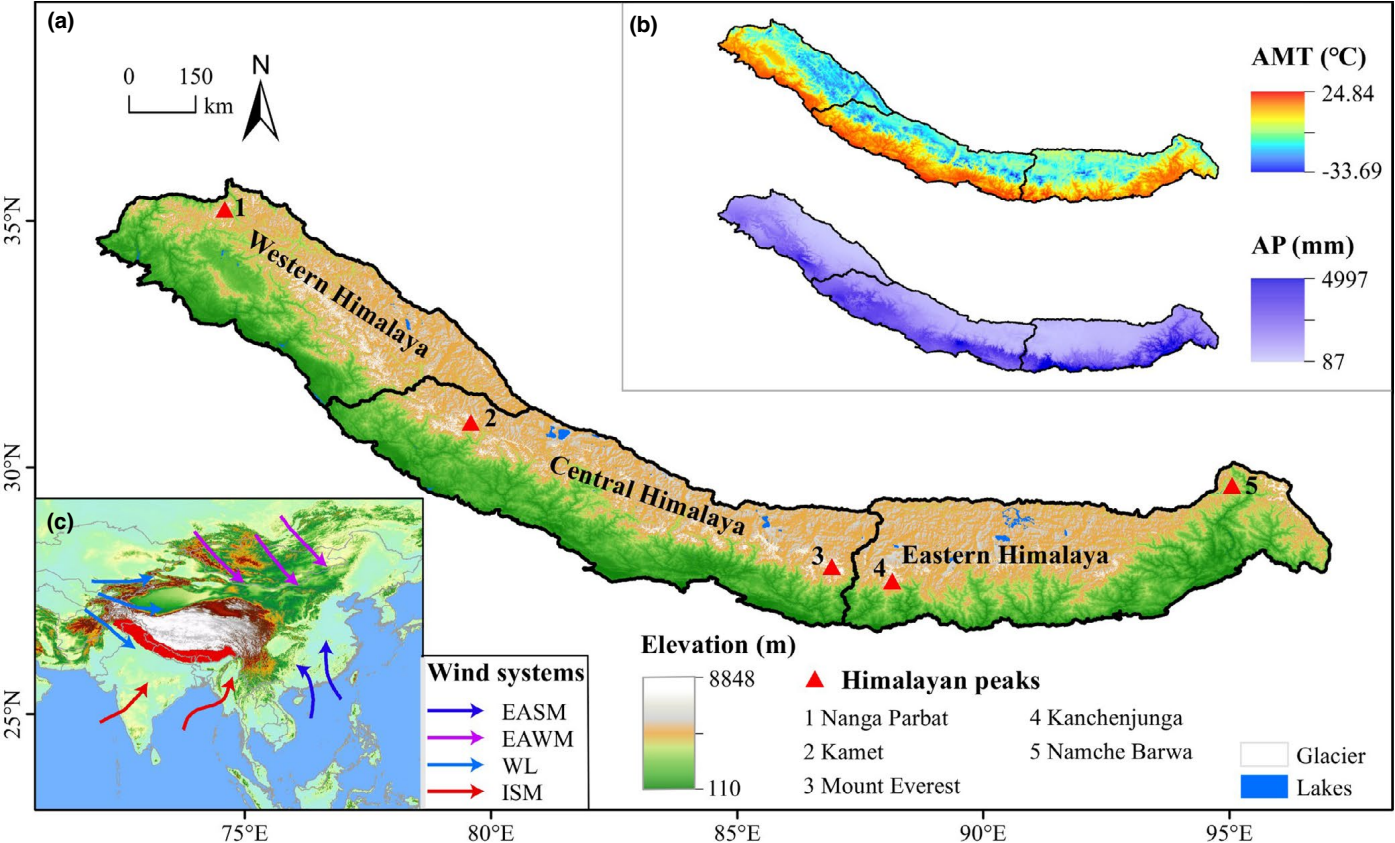
Geographical and climatic characteristics of the Himalaya. (a) Topographical map of the Himalaya, onto which the boundaries of western, central, and eastern Himalaya were newly drawn according to Bolch et al. (2012) and Apollo (2017). (b) The annual mean temperature (AMT) and annual precipitation (AP) across Himalaya; the climatic variables were downloaded from WorldClim (Fick et al., 2017). (c) Geographical location of Himalaya and the atmospheric circulation systems in East Asia according to An et al. (2014); the arrows indicate the wind systems. EASM, East Asian summer monsoon; EAWM, East Asian winter monsoon; WL, Westerlies; ISM, Indian summer monsoon

With a geographical area of more than 600,000 km^2^, the Himalaya comprises a series of mountain ranges that span the boundaries of five nations: China, India, Bhutan, Nepal, and Pakistan (Figure 1a). In geological terms, the Himalaya comprises four distinct tectonic units: the Outer Himalaya (or Siwaliks), Lesser Himalaya, Greater Himalaya, and Trans‐Himalaya (Valdiya, 2002). With its highest peak (Mount Everest) measured to be 8,848.86 m (http://www.xinhuanet.com/english/2020‐12/08/c_139573391.htm), the Himalaya is the highest mountain system on Earth and has a complex orogenic history, but one riddled with controversy (Currie et al., 2005; Ding et al., 2014; Spicer, 2017). However, the consensus is that the uplift of the Himalaya is intertwined with the geological events that led to the formation of the Tibetan Plateau and the surrounding mountain ranges. The Plateau was formed following the collision of the Indian and Asian continents *ca*. 55–50 ± 10 Ma (million years before present) (An et al., 2021; Klootwijk et al., 1992; Yin & Harrison, 2000; Zhang et al., 2012), and by the late Miocene (8–10 Ma), much of the Himalaya had attained its current elevation (Ding et al., 2017; Garzione et al., 2000; Garzione et al., 2000; Rowley et al., 2001; Spicer, 2017). However, a recent synthesis by Spicer, Su, et al. (2020) cast doubt upon this simplistic model of plateau formation and instead argues for a more complex process that involved earlier collisions of Gondwanan terranes with Asia, forming a high relief landscape before the arrival of the Indian subcontinent. A recent study constrained the middle Eocene height of a valley in Central Tibet to ~1,500 m (Su et al., 2020), while the proto‐Himalaya started building against the pre‐existing Gangdese highland along the southern margin of what would later become the Tibetan Plateau. Radiochemical and fossil evidence indicates that the proto‐Himalaya began to grow against the then ~4,000 m‐high Gangdese Mountains from ~1,000 m in the late Paleocene to ~2,000 m in the early Miocene, with some parts attaining about 5,000 m by around 15 Ma (Ding et al., 2017), and that Mount Everest area had already attained ≥5,000 m by the early Miocene (Gébelin et al., 2013).

**FIGURE 2:**
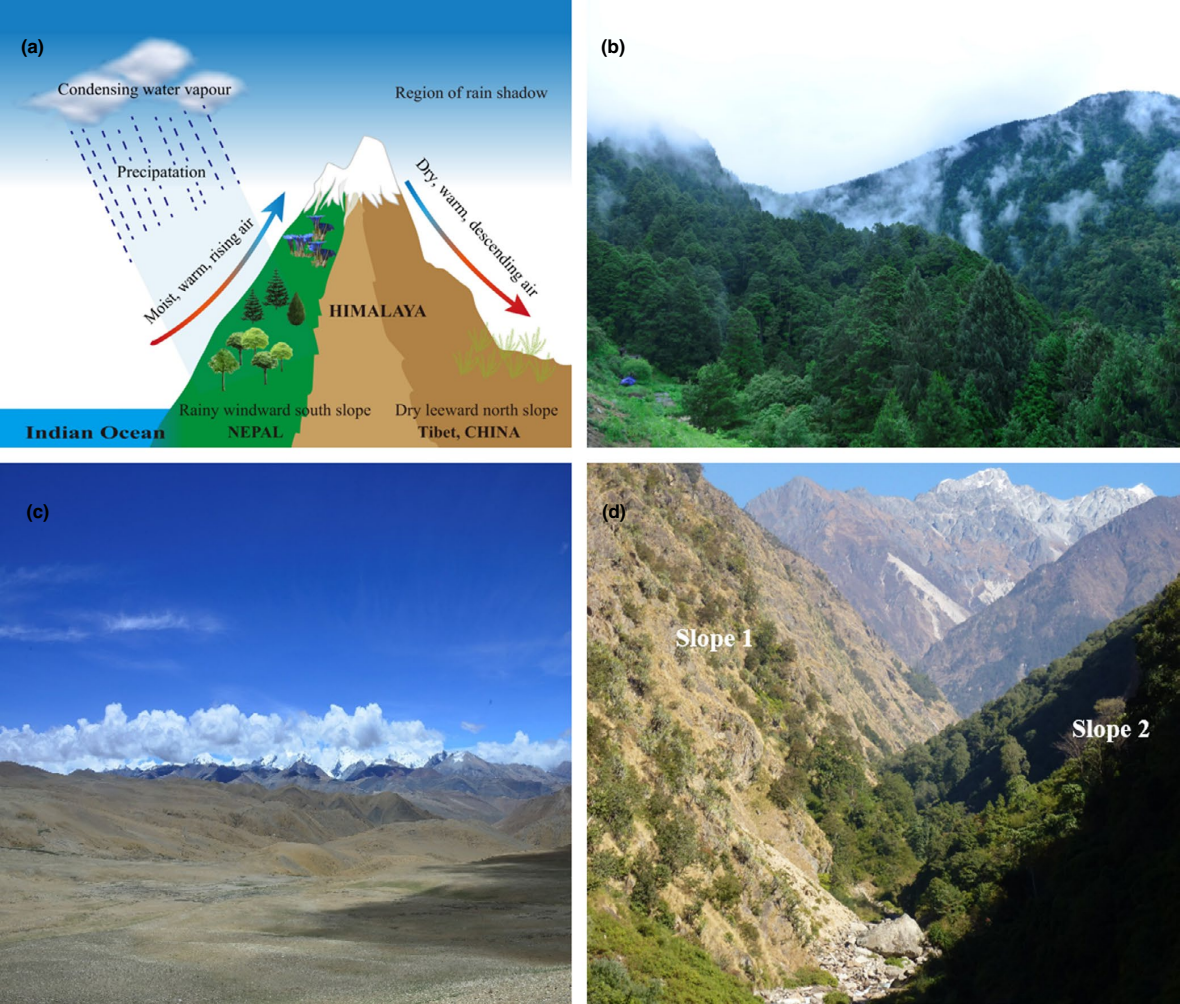
Geoclimatic features of the Himalaya. (a) A schematic model showing topography, the Indian summer monsoon, and its associated precipitation, plus rain shadow in the central Himalaya (e.g., NEPAL‐CHINA section). The Himalaya blocks moist air, leading to increased precipitation on the windward southern slope and a dry climate on the leeward northern side. Consequently, the two slopes of the mountain differ in terms of vegetation type, as well as species diversity, along their elevational gradients. (b) and (c) respectively show representative photographs of the southern windward and the northern leeward sides of the Himalaya range. (d) Depiction of slope and aspect as community differentiators in the East‐West trending Langtang Valley, Nepal. The south‐facing slope is hot and dry (Slope 1), while the north‐facing slope is cool and moist (Slope 2), leading to habitat heterogeneity over short distances

The Himalaya and its surrounding landscape have attracted immense research interest particularly from biologists and climatologists, largely because of the idea that the Himalaya–Tibetan orogeny is a principal driver of the Asian monsoon system (Figure 1c). This is particularly so for the South Asia monsoon (Indian summer monsoon), which dominates current regional climate dynamics, and has profoundly affected the evolution and diversification of regional flora (Spicer, 2017; Wang et al., 2012). Generally, the Himalayan climate is highly variable over short distances owing to the topographic heterogeneity of the region (Figures 1a, 2). Climatically, the Himalaya can be divided into Western, Central, and Eastern regions (Apollo, 2017; Bolch et al., 2012). The Eastern and Central Himalaya, which harbor the highest species richness, are usually characterized by a warm and wet climate owing to the moisture‐laden air supplied by the Indian summer monsoon, while the western sector has a warm and dry climate (Figure 1). The general climate of the Himalaya is also influenced by the effects of two other wind systems, that is, the East Asian winter monsoon and the Westerlies (Figure 1c). The Himalaya generally exhibits an east‐west orientation, where the windward southern sides experience rainy and warm conditions, whereas the leeward northern sides by contrast have dry and cold climates (Figures 1b, 2a, b, c). However, individual valley systems show a different pattern, in which the south‐facing slopes are hot and dry, while the north‐facing slopes are cool and moist (Figure 2d). This illustrates the incredible environmental heterogeneity that can exist over short distances, and may help to explain the high frequency of narrow endemics in the Himalaya, though the underlying mechanism would need clarification.

The Himalaya and its surroundings host several of the world's key biodiversity hotspots (Mittermeier et al., 2004; Myers et al., 2000), and the region is more affected by climate change than anywhere outside of the Arctic and Antarctic (Liu, Milne, Cadotte, et al., 2018). Rapid climate change in the Himalaya continues to pose a threat to the survival of many species, and this effect has been exacerbated by the intensification of anthropogenic activities such as deforestation and urbanization, and these have led to significant land‐cover changes (Cui & Graf, 2009; Liu, Milne, Cadotte, et al., 2018). Indeed, recent studies and syntheses have increasingly implicated human activities as the main drivers of changes in biodiversity, at both gene and species levels (e.g., Emel et al., 2020; Woodbridge et al., 2020). However, accelerated ice loss over the last four decades (Bolch et al., 2012; Maurer et al., 2019), particularly in the drier western Himalaya (Lutz et al., 2014) has also significantly disrupted ecosystem services in the Himalaya. The melting of the glaciers in the Himalaya is caused primarily by rising air temperatures, which have risen by 1℃ since 1975, much faster than the global mean, which has risen by 0.84℃ since 1,880 (IPCC, 2013; Maurer et al., 2019). These disturbance factors together could mean a higher extinction risk for Himalayan species (Pandit et al., 2014).

**FIGURE 3:**
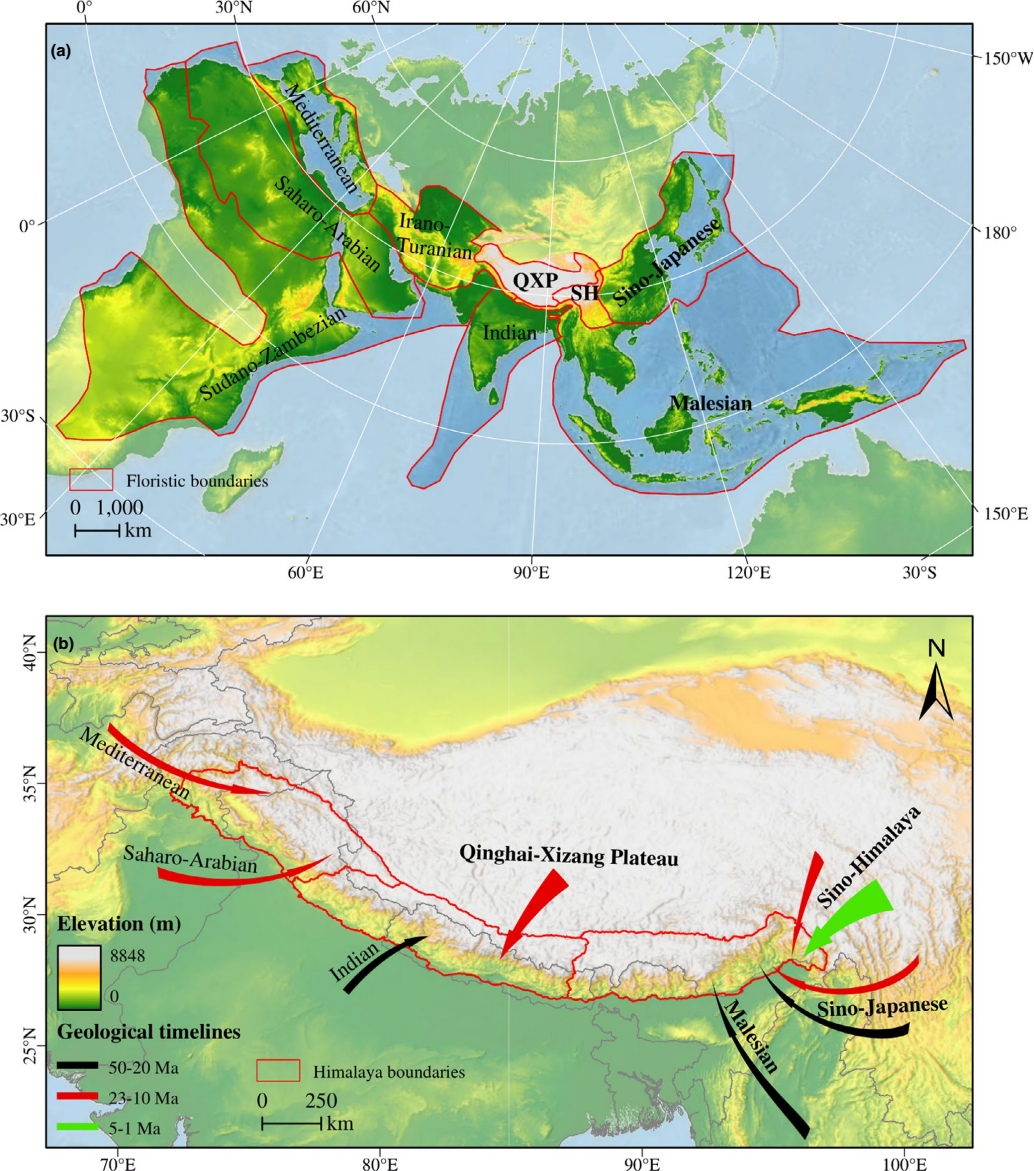
Floristic boundaries and immigration of flora into the Himalaya. a) Estimated boundaries of the key floristic divisions surrounding the Himalaya; subkingdom names are shown in bold font while region names are in regular font. Boundary demarcation is based on Takhtajan (1986; Indian, Irano‐Turanian, Mediterranean, Saharo‐Arabian, and Sudano‐Zambezian floristic regions) and Wu and Wu (1996; Malesian, Sino‐Japanese, SH, and QXP subkingdoms). b) Major patterns of early (pre‐Pleistocene) floristic colonization of the Himalaya from different regions. The first wave (black arrows) occurred mainly in the Eocene and Oligocene (about 50–30 Ma; Manish & Pandit, and references therein), while the second wave (red) occurred from early to Mid‐Miocene (23–10 Ma; Mani, 1974; Mehrotra et al., 2005). Following the increased topographic heterogeneity in the Hengduan Mountains in the late Miocene (around 8 Ma), there was increased diversification and subsequent dispersal of species from the Hengduan Mountains into the Himalaya (green arrow; Xing & Ree, 2017). Arrow sizes are proportional to the number of migrating species (estimated from Ding et al., 2020; Singh & Singh, 1987). SH, Sino‐Himalaya; QXP, Qinghai–Xizang Plateau

The floristic patterns of the Himalaya appear to generally conform to its geophysical attributes, confirming the dominant role of geological and climatic factors in shaping the contemporary distribution of the regional flora. According to Takhtajan (1986), the Himalaya falls within two floristic kingdoms: Palaeotropical Kingdom (eastern Himalaya) and Holarctic Kingdom (western Himalaya). Likewise, at a more local scale, the Eastern and Western Himalaya belong to separate floristic regions, namely the Eastern Asiatic and Irano‐Turanian regions, respectively (Takhtajan, 1986). However, a subsequent revision elevated Takhtajan's Eastern Asiatic Region to Kingdom status, in which the Eastern Himalaya and Western Himalaya were treated as floristic regions, but with these defined regions belonging to different subkingdoms: Sino‐Himalayan, Qinghai–Xizang Plateau, and Malesian Subkingdoms (Wu & Wu, 1996) (Figure 3a).

Here, we review the origins of floral elements and the consequences of past and future climate change in the Himalaya and then discuss the implications of such change for conservation strategies. Although many aspects of Himalayan biodiversity have been addressed in previous reviews, gaps still remain regarding the current state of knowledge about effects of future climate changes on biodiversity. We therefore highlight key areas of debate and pinpoint the outstanding research questions that remain unexplored. Furthermore, as most of the studies in the Himalaya region have focused on a unified Himalaya–Hengduan Mountains or Tibetan Plateau (sensu *lato*) complex, our review will make occasional reference to the mountain systems surrounding Himalaya, but attempt to center most of the discussion on the Himalaya proper. We end the review by recommending a raft of tools and approaches that might be useful for future research on Himalayan biota.

## 2 BIOGEOGRAPHY OF THE HIMALAYAN FLORA

### 2.1 Floral assembly through immigration

It is estimated that the Himalaya supports more than 10,000 species of vascular plants, with almost a third (31.6%) being endemic (Mittermeier et al., 2004), although the precise number of species remains controversial, and many new plant species continue to be described from the region (e.g., Borah et al., 2020; Maity, 2014). The relatively high endemicity is thought to be a product of in situ diversification of immigrant species from outside of the Himalaya following the uplift of the region and the subsequent climatic upheavals (Manish & Pandit, 2018). Although the precise mechanisms and drivers of the dispersal, colonization, and diversification processes are still a matter of ongoing research, certain events seem clear. Early on, before the collision of the Eurasian and Indian plates and the subsequent uplift of the Himalaya, a biological vacuum seems to have existed across much of the southern margin of Eurasia due to widespread aridity (Farnsworth et al., 2019 and references therein). Although it would be a difficult task for future investigations to quantify Paleogene diversity in the Himalaya region, such information (if available) would further strengthen the existing evidence for postextinction colonization and clarify the patterns thereof. The biological vacuum was later filled through large‐scale floral migrations from multiple directions, with Euro‐Mediterranean affinities mostly colonizing the western region. The first arrivals in the east might have been taxa with tropical elements of Sino‐Japanese and Malesian affinities (Manish & Pandit, 2018, and references therein; Figure 3b), some of which later dispersed to the northeastern Himalaya via Tibet and Kashmir (Mehrotra et al., 2005). These first arrivals, then, gave rise to the flora that was present as significant uplift began. East‐west orientated ranges such as the Himalaya tend to present greater topographical barriers toward north‐south migration in response to Pleistocene climate cycles, relative to north‐south orientated ranges like the Hengduan Mountains. This, coupled with the Hengduan Mountains having experienced relatively less glaciation (Shi et al., 2006), means that extinction rates in the Himalaya would likely have been higher. However, these factors might have had a lesser extinction effect on the southern slope of the Himalaya range, as species there could have easily migrated southward and downhill unrestricted during the cooler epochs.

There is increasing evidence that the Himalaya's current floristic elements mostly originated from the east and west (Cun & Wang, 2010; Liu et al., 2013, 2018; Rana et al., 2019; Singh & Singh, 1987; Xing & Ree, 2017; Yu et al., 2015) and possibly also via the pre‐existing Gangdese (Spicer et al., 2020). Most of the floristic exchange appears to have been localized between the eastern Himalaya and the Hengduan Mountains, although certain taxa of Euro‐Mediterranean origins did colonize the Western Himalaya (Figure 3b). Based on a multitaxa approach, Xing and Ree (2017) demonstrated that immigration contributed more to biotic assembly in the Himalaya than in situ speciation, thus concluding that most of the extant plant species in the Himalaya arrived there by colonization in the late Miocene. Likewise, Yu et al. (2020) proposed that immigration might have been more important than in situ diversification in the Himalaya, with mostly the Hengduan Mountains serving as a major source (also Ding et al., 2020; Figure 3b). Increased rates of in situ diversification in the Hengduan Mountains (*ca*. 8 Ma) predate the onset of dispersal events between the Hengduan Mountains and Himalaya–Tibet (*ca*. 4–5 Ma) (Xing & Ree, 2017), which lends support to the recent finding that most of the alpine Himalayan floral elements were derived from the Hengduan Mountains in the east (Ding et al., 2020). However, these inferences about floral origin were based on where taxa are found today—not where they may have been in the past, hence the conclusions may need a re‐examination using fossil evidence. For instance, it is important to consider the role of the Gangdese paleofloral elements from the middle Miocene, as some of these taxa and their relatives are known to exist in the Himalaya today (Guo et al., 2019). However, the contribution of the Gangdese, whose paleofloral and current species compositions are very different, can only be explored using fossil evidence; use of molecular evidence alone in such a case would bias the conclusions about taxon origin and point only to the regions where the related taxa live today. A consideration of both lines of evidence (molecular and fossil data) would therefore provide clearer insights into the spatiotemporal origin of plant taxa in the region.

Phylogeographic investigations support this trend of biodiversity assemblage through immigration. One such study supported a westward range expansion of the perennial herb *Triosteum himalayanum* from the Hengduan Mountains to the eastern Himalaya during the last interglacial (Liu, Gao, et al., 2018). Moreover, Cun and Wang (2010) suggested a pre‐LGM (Last Glacial Maximum; *ca*. 21 Ka, thousand years before present) recolonization of the Himalaya by the wind‐dispersed Himalayan Hemlock *Tsuga dumosa* from the Hengduan Mountains refugium. This westward immigration is supported by the idea that the Hengduan Mountains flora is older than that of the Himalaya, with the earliest diversification events occurring by 64 Ma for the Hengduan Mountains and 59 Ma for the Himalaya (Xing & Ree, 2017). By contrast, a limited number of single‐taxon investigations present evidence for dispersal in the reverse direction (Himalaya to Hengduan Mountains) (Rana, Luo, et al., 2019; Zhou et al., 2013). Other than the Hengduan Mountains, elements of the Himalayan flora have also been derived from the area that is now the Tibetan Plateau sensu *stricto*, although the degree of floristic exchange between the two areas is relatively low (Ding et al., 2020; Yu et al., 2020; Figure 3b). Such dual origins of montane biodiversity are not limited to the Himalaya; similar phenomena have been demonstrated for Mount Kinabalu in Borneo based on a large range of organisms, but with a majority of organisms deriving from local ancestors at lower elevations on the same island (Merckx et al., 2015). This hints at the role of altitudinal gradients, as well as the interplay between long‐distance dispersal and local recruitment, in driving the evolution and speciation of montane biota, and this could also be true for the Himalaya.

### 2.2 In situ diversification

In spite of the major role played by immigration in the assembly of the Himalayan flora, numerous studies have argued in favor of in situ speciation and diversification, which were triggered by the orogeny and the subsequent climatic changes in the Himalaya and its surrounds (e.g., Bai et al., 2015; Rana, Luo, et al., 2019; Ren et al., 2017; Xie et al., 2014; Xing & Ree, 2017; Zhao et al., 2016), and which could offer a convincing explanation for the high endemism in the Himalaya. Indeed, alpine radiations in the Himalaya generated relatively more species than those in other major mountain ecosystems (Hughes & Atchison, 2015). Recently, Ding et al. (2020) demonstrated that accelerated in situ alpine diversification (observed as peaks in diversification rate plots) in the Himalaya coincided with periods of rapid uplift and intensification of the Asian summer monsoon. Manish and Pandit (2018) had earlier outlined five phases of Himalayan geophysical and/or climatic upheavals, each of which can be linked to local diversification of plant species. The first phase, which constituted the mega‐collision of the Indian and Eurasian plates 61 Ma (An et al., 2021), led to the formation of land bridges that in turn facilitated the migration and diversification of plant species (Pandit, 2017). Fossil data show that the first migrants into what is now the Eastern Himalaya region arrived there soon after the collision of Indian and Eurasian plates (Klaus et al., 2016; Mehrotra et al., 2005; Figure 3b). The advantage of the fossil record is that it documents the location in time and space of extinct organisms but it is, by its very nature, incomplete as only a small fraction of organisms alive at any one time become fossilized. Previous studies have generally been based on these rare occurrences (Deng et al., 2019; Ding et al., 2017; Spicer et al., 2017; Srivastava & Mehrotra, 2010; Su et al., 2020). Other than finding more fossils, there are several ways by which the record of past biome assemblies can be improved. These include integrating plant and animal fossil data, combining the fossil records with molecular phylogenies that draw on the history of genomes that have survived to the present and integrating such data with numerical models of biodiversity driven by palaeoclimate models (e.g., Li et al., 2021).

The subsequent tectonic uplift and landform elevation of the Himalaya created more land area experiencing cooler climate conditions in the region, thereby favoring the second wave of immigration, which mainly involved certain temperate taxa from the Sino‐Japanese and Euro‐Mediterranean regions between 20 and 10 Ma (Mehrotra et al., 2005; Figure 3b). This mechanism is consistent with the Mountain‐Geobiodiversity hypothesis (Mosbrugger et al., 2018; Muellner‐Riehl et al., 2019), which proposes that factors such as geodiversity evolution and Neogene/Pleistocene climate changes may have jointly contributed to the evolution of mountain biodiversity. A few studies have demonstrated this influence of Himalayan uplift on species dispersal and diversification (e.g., Meng et al., 2017; Xie et al., 2014; Zhao et al., 2016) with the evidence mainly indicating Miocene divergence. For instance, the divergence of two Himalayan alpine ginger genera (*Cautleya* and *Roscoea*) took place between the middle Eocene and early Oligocene, a timeline that corresponds with widespread regional tectonism and the rise of several parts of the Tibetan region sensu *lato* (Li et al., 2020; Spicer, Farnsworth, et al., 2020; Su et al., 2019; Xiong et al., 2020). The divergence of two major clades within *Meconopsis* (*Meconopsis* and *Meconella* clades) was also attributed to these major uplift events of the Himalaya, and the initiation of monsoonal climates in the region around the early Miocene (20–15 Ma). However, some of the conclusions in these studies may require re‐examination. First, there is increasing evidence that monsoonal climates in the region have a much longer history (Spicer, Farnsworth, et al., 2020; Spicer et al., 2017, and references therein), and what Xie et al. (2014) refer to as “initiation” could just be changes in certain monsoon features, such as seasonal differences in rainfall due to factors such as global mean temperatures and topography. Secondly, the conclusion attributing diversification to tectonic uplift was based on the earlier assumption that the Himalaya–Tibet was uplifted uniformly as a coherent unit, a view that was recently questioned by Spicer, Su, et al. (2020). Thirdly, most studies that tested the hypothesis that Himalayan uplift promoted plant speciation tended to use taxa with wide distribution ranges, covering the entire Himalaya–Tibetan region. Such large spatial scales may have confounded the actual evolutionary dynamics within the Himalaya proper. It would be desirable for future investigations to lay more emphasis on Himalayan endemics, while concurrently integrating geodiversity parameters with Neogene/Pleistocene climatic variables in the studies, to specifically test the “Mountain Geobiodiversity Hypothesis” in the Himalaya (Mosbrugger et al., 2018; Muellner‐Riehl et al., 2019).

The third phase of upheaval and diversification involved the onset and subsequent intensification of the Indian summer monsoon, which has been linked for example to the divergence of *Parapteropyrum tibeticum* from *Fagopyrum* about 15–6 Ma (Tian et al., 2011). Moreover, a recent radiation in *Maianthemum* (*ca*. 2.4 Ma) overlapped with the period of extensive climatic changes in the eastern Himalaya (Meng et al., 2008). Furthermore, the early diversification and subsequent radiations in the fern genus *Lepisorus* were found to be driven by the intensification of the East Asian monsoon in the late Miocene and Pliocene (Wang et al., 2012). These examples suggest that climatic changes associated with monsoonal evolution, particularly the late Neogene intensification (Farnsworth et al., 2019), might have imposed new climatic barriers that subsequently drove the Miocene/Pliocene diversification in the Himalaya region. However, Renner (2016) rightly advises caution when ascribing any post‐Miocene radiations to the initiation of monsoonal system, as geological data have demonstrated that monsoon systems predate the Miocene, and may have existed throughout most of Earth's history (Spicer et al., 2017; also discussed above). In fact, a monsoon system similar to the Indian and East Asia summer monsoons may have existed as far north as Central Tibet in the middle Eocene (Su et al., 2020). The Miocene radiations in the region, therefore, might have, at best, weak links with the monsoon system (except in the case where a pre‐existing monsoon undergoes changes), suggesting that orogenic factors generating complex niche patterns could be the main drivers of the diversification during this time.

The fourth phase, following the intensification of the Asian summer monsoon system, was the increase in aridity in Central Asia, due to the obstruction of moisture‐carrying winds by the elevated Himalaya and central Tibet (Figure 2a) (Miao et al., 2012). This increased aridification prompted drought‐ and cold‐tolerant lineages to both colonize the newly forming Tibetan Plateau and diversify there; for example, the three main lineages of *Ephedra* diverged during the middle or late Miocene (about 10–5 Ma) (Qin et al., 2013).

Quaternary climate oscillations (since 2.5 Ma) have also been linked to the evolution and divergence of Himalayan species (Khan et al., 2017; Meng et al., 2008; Wallis et al., 2016; Wang et al., 2016), marking the fifth and final phase of abiotically driven diversification in the region. This chronology of events is consistent with the argument of Muellner‐Riehl (2019) that diversification of montane plant species is not only driven by orogenesis, but also by a complex interaction of factors such as topography and climate. Consistently, Spicer, Farnsworth, et al. (2020) proposed that during glaciations, previously isolated species might have migrated to lower elevations and subsequently hybridized to form new species.

The examples outlined above highlight the role of the interplay between topographic and climatic factors in driving the floral assembly and diversification of plant species in the Himalaya. Almost invariably, this interaction involves both spatial (horizontal and vertical) and temporal scales through geographic isolation and climate changes from past to present (Spicer, 2017). Despite the importance of abiotic factors, it is likely that certain biological factors could be more important drivers of the distribution and diversification of some species. For instance, ancient polyploidization and hybridization, as well as the distribution patterns of pollination or dispersal agents (and their respective pathogens), may influence the distribution patterns of plant species. Future studies should explore the possibility of using such biotic factors as proxies for understanding the biogeographical history plant species.

### 2.3 Barriers to gene flow and dispersal corridors

One of the key drivers of biogeographic patterns in the Himalaya is the existence of local geophysical barriers. Poudel et al. (2014) identified two strong barriers to gene flow in *Taxus*, both of which were attributed to Himalayan mountain ranges: one to the north (Dhaualagiri‐Annapurna‐Manaslu range) and the other to the south (Mahabharat range). Furthermore, the entire Himalayan mountain system itself has been shown to act as a barrier to north‐south gene flow, for example, in the wind‐pollinated shrub *Hippophae tibetana* from Nepal and Tibet (Qiong et al., 2017), though the difference in climate between the two sides of the range may also contribute to the genetic differentiation. Local topography (Himalayan ridges and peaks) and habitat heterogeneity were recently found to be significant barriers to gene flow in *Incarvillea arguta*, although variation in monsoonal circulation patterns appeared to confound the effect of these topographic barriers (Rana, Luo, et al., 2019). Additionally, a climatically stable, long‐lasting 500‐km‐wide ecological barrier (characterized by relatively low temperatures and high precipitation) between the Himalaya and the Hengduan Mountains has been identified (Li et al., 2019) and demonstrated to have a key role in maintaining the disjunct distribution of *Roscoea* between the two regions. However, studies on the evergreen tree *Juniperus tibetica* (Opgenoorth et al., 2010) and the Himalayan hemlock *Tsuga dumosa* (Yu et al., 2015) did not find any obvious geographical barriers along their putative dispersal pathways. These studies highlight the fact that although geographical barriers in the Himalaya region play a role in diversification of the regional flora, the varied response among species calls for more studies in order to test the specific effects of life form, pollination/dispersal mode, and vagility. Moreover, gaps in understanding still exist with regard to the integration of geographical barriers with anthropogenic forces to shape the distribution and genetic structure of plant species in the region. In order to gain meaningful insights into the interplay of these factors, studies should examine multiple species over wider spatiotemporal scales.

Although some studies have demonstrated a lack of obvious physiographic barriers in the Himalaya (Opgenoorth et al., 2010; Yu et al., 2015), specific hypotheses should be tested to examine the presence and role of climatically imposed barriers. Liu et al. (2013) showed that genetic differentiation between two *Taxus wallichiana* lineages, from the Eastern Himalaya and the Hengduan Mountains, was mainly attributable to differences in precipitation. Similarly, lineage diversification in *Incarvillea arguta* is thought to have been driven by climatic differences in the Himalaya‐Hengduan Mountains region (Rana, Luo, et al., 2019). Although these studies only report intraspecific lineage divergence, they demonstrate the potential of climatic barriers as drivers of speciation in the Himalaya. In spite of this potential, the extent to which climatic factors would act as genetic barriers, independently of orogenesis, is unclear, as studies on the topic have tended to explain the observed climatic differentiation in terms of orogenic history. The recent advancements in geospatial analytical tools and the accessibility of refined climatic data provide an unprecedented opportunity to delineate the relative contributions of climatic forces and orogenic factors in shaping the distribution and genetic structure of species in the Himalaya.

With that in mind, geological and climatic forces also seem to have worked in concert to aid dispersal and intraspecific gene flow in Himalayan plant species. For example, Yu et al. (2015) identified the southern slopes of the Himalaya as dispersal corridors based on a combination of coalesced estimates of migration rates and genetic divergence patterns of the Himalayan hemlock (*Tsuga dumosa*). This southern dispersal corridor has allowed *T*. *dumosa* to migrate from the eastern Himalaya onto the margins of the Tibetan Plateau, particularly during the last few decades (since ~1950). More recently, drainage systems, mountain gorges, and valleys were identified as key dispersal corridors for *Incarvillea arguta* (Rana, Luo, et al., 2019) and *Mirabilis himalaica* (Rana et al., 2020). Despite the useful insights from these studies, a meta‐analysis (based on multiple species) would be required to reveal more general patterns and possibly identify more dispersal corridors in the region.

### 2.4 Elevational gradients and species diversity

**FIGURE 4:**
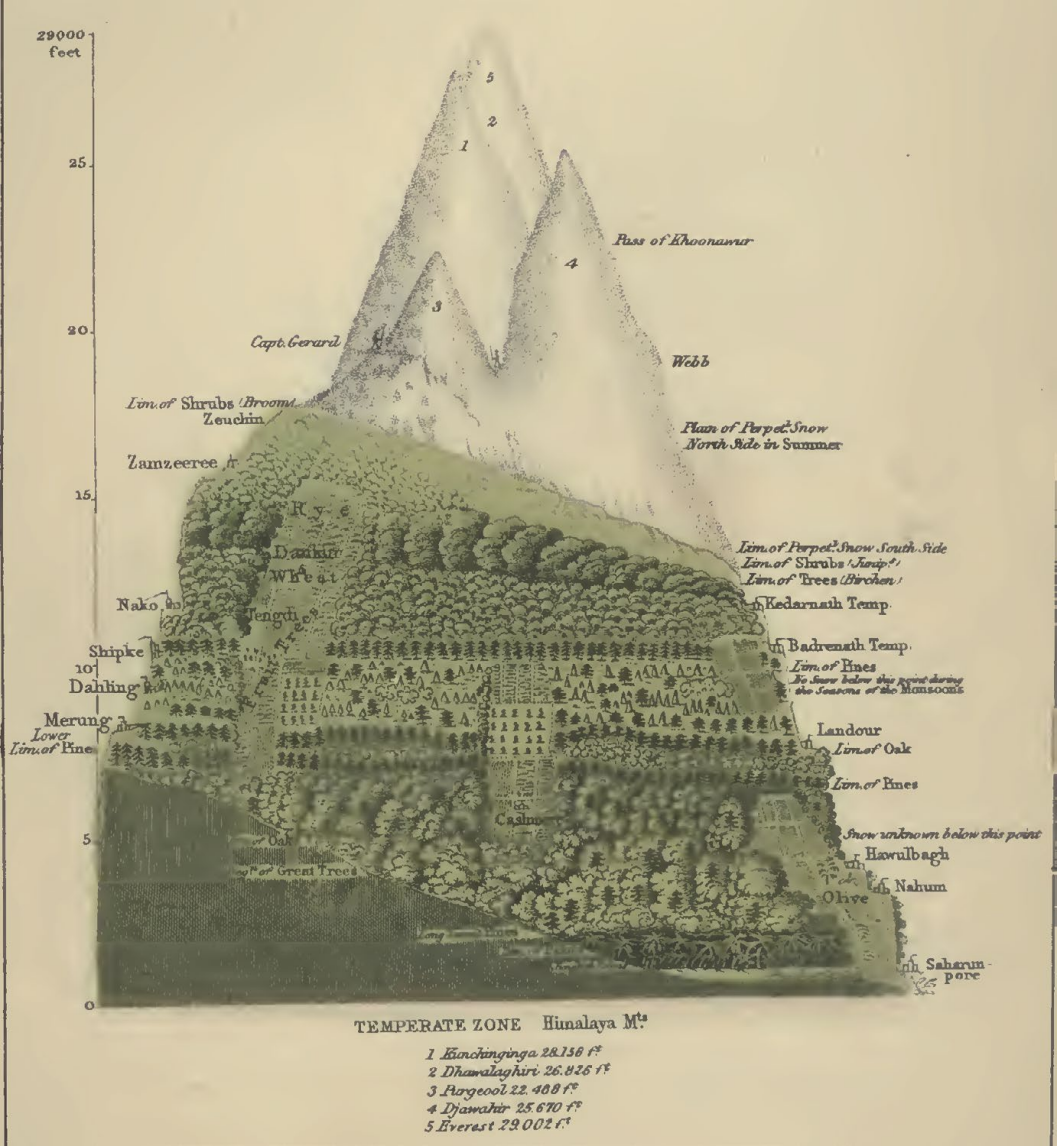
Illustration of plant vertical distribution along the elevational gradient of the Himalaya by Hughes (1866). From the Philips' Atlas of Physical Geography, 1866 (Hughes). A digital version of this book is available online from HATHITRUST and INTERNET ARCHIVE

The most fascinating aspect of the Himalaya is the huge elevational range from nearly sea level to an excess of 8,000 m. For this reason, most of the investigations on species diversity in the Himalaya have been conducted in the context of elevational gradients, which potentially drive diversification and speciation (e.g., Caro et al., 2013; Li et al., 2014). Research interest in Himalayan elevational gradients dates back over a hundred years, as shown by Hughes (1866) who illustrated that different elevational belts were dominated by different types of vegetation (Figure 4), due to the climate‐dependent niche heterogeneity on mountain slopes. The role of such contemporary environmental gradients may however be confounded by the combined effect of Cenozoic topographical and climatic changes, which impacted the elevational distribution of most taxa (Spicer, Farnsworth, et al., 2020, and references therein). A case in point is *Capparis spinosa*, which underwent range contractions at high altitudes during the LGM following the onset of cold climates associated with advancing glaciers in the western Himalaya, but with low‐altitude populations experiencing minimal effects from the glaciation (Wang et al., 2016).

Speciation events in montane ecosystems are often driven by steep changes in the biotic and abiotic factors along altitudinal and environmental gradients, particularly in light of altitudinal hybrid zones, barriers to gene flow, and local adaptation (Abbott & Brennan, 2014; Caro et al., 2013; Filatov et al., 2016; Segar et al., 2016). In this speciation scenario, altitudinal gradients lead to differing microclimatic conditions between different elevations, resulting in population differentiation through the interplay of adaptive and neutral evolutionary processes (De Villemereuil et al., 2018). Although several studies in the Himalaya have attempted to address the question of speciation mechanisms and their timing (e.g., Liu et al., 2013; Xie et al., 2014), the role of altitudinal gradients as drivers of Himalayan speciation still remain largely unexplored. Future studies should explore ecological speciation mechanisms, particularly those driven by the niche partitioning of closely related species along the elevation. On the same note, the role of thermal belts in floral assembly along elevational gradients needs to be addressed in detail, as explored recently in other mountain ecosystems (Griffiths et al., 2021; Molina‐Venegas et al., 2020).

The Himalaya provides a unique opportunity to evaluate species diversity patterns and their origins in a complex landscape. Literature on the relationship between elevational gradients and species richness in the Himalaya is available for liverworts and mosses (Grau et al., 2007), ferns (Bhattarai et al., 2004), gymnosperms (Subedi et al., 2019), and flowering plants (Bhattarai et al., 2004; Rana et al., 2019; Wang et al., 2007). Both unimodal and bimodal relationships between species richness and elevation have been commonly observed in various plant taxa (Acharya et al., 2011; Ahmad et al., 2020; Bhattarai et al., 2004; Kluge et al., 2017; Oommen & Shanker, 2005; Sun et al., 2020). Moreover, endemic species richness in the region appears to peak at markedly higher elevations than total species richness (Kluge et al., 2017; Vetaas & Grytnes, 2002), possibly because climate change (especially historical temperature fluctuation) is greater at high elevations; hence, there are more perturbations at these high elevations that in turn drive speciation. Despite the variation in detailed relationship patterns, both unimodal and bimodal patterns highlighted above indicate a midelevation peak (~2,000–4,000 m; subalpine to alpine but not extreme high alpine) in species richness, though a recent study in the east Himalaya suggested a low‐elevation peak (500–1,000 m) (Rana et al., 2019). Higher elevations might tend to have fewer species because of the extended Rapoport's rule (Körner, 2007): Area is positively correlated with species richness, but decreases with increasing altitude. Low elevations, however, have the highest level of anthropogenic activities, and if this depresses species richness then these effects together would explain the midelevation peaks. This explanation agrees with the theory that the hump‐shaped relationships between species richness and elevation arise from random occurrences of species that have different geographical ranges that overlap between two ecologically unfavorable boundaries (i.e., lowlands and mountain tops) (Brehm et al., 2007). In addition, the extreme ends of an elevational gradient might have experienced more drastic selection pressure during climatic fluctuations in the past, particularly if the overlap between current and LGM temperature profiles is strong (Muellner‐Riehl et al., 2019).

In general, temperature‐ and water‐related variables appear to be the key determinants of species richness in mountain ecosystems (Antonelli et al., 2018; Steinbauer et al., 2018), although their effects might be higher in herbaceous plants than in woody taxa (Lu et al., 2018). Based on a comprehensive dataset of 137 plant families in the Himalaya, Manish (2019) identified elevational gradients as one of the key drivers of species diversity and showed that temperature was the best explanatory variable for the observed elevational variance in species richness. Furthermore, Rana, Price, et al. (2019) found mean temperature and annual precipitation to correlate with about 60% of the variation in angiosperm species richness in the Himalaya. For example, the distributional range of a Himalayan treeline species *Betula utilis* was found to be primarily associated with thermal and precipitation‐related factors (Bobrowski et al., 2017). The joint importance of temperature and water as a predictor of species richness along Eastern Himalayan elevational gradients has also been demonstrated (Acharya et al., 2011; Devkota et al., 2019), but hypotheses on the effects of specific water‐related factors (precipitation, humidity, and soil moisture) need to be untangled. Since temperature and precipitation have also been closely associated with the increased rate of glacier melting in the Himalaya (Kraaijenbrink et al., 2017), this link could represent a potentially fruitful area of research. In a recent review, Cauvy‐Fraunié and Dangles (2019) argued that the empty niches created by retreating glaciers can act to reduce competition among species, as the most efficient dispersers would colonize the new niches, thus increasing between‐habitat connectivity. It would be worthwhile for future investigations to explore whether the biotic and abiotic pressures in the newly colonized niches would favor diversification and subsequent speciation.

Beta diversity, which quantifies variability in community composition among sampling units (Whittaker, 1960), is increasingly becoming important for understanding the mechanisms of biodiversity maintenance (e.g., Baselga, 2010; Condit et al., 2002; Du et al., 2021). Although beta diversity patterns of plant communities in the Himalaya have received less attention than alpha diversity, previous studies indicate that topographic (e.g., slope orientation and elevation), environmental (e.g., soil properties), and anthropogenic (land use) factors are the key determinants of beta diversity patterns in the region (Paudel & Vetaas, 2014; Rawat et al., 2020). This subject should be seriously considered in future research, particularly regarding the effect of species turnover among phylogenetic relatives that share similar functional traits. Thus far, one study of phylogenetic beta diversity has detected strong evidence of lineage filtering, but not clear trends of phylogenetic relatedness, along eastern Himalayan elevational gradients (Shooner et al., 2018). Furthermore, a triphasic pattern (decrease–increase–decrease) of phylogenetic dispersion in angiosperm assemblages was observed along an elevational gradient within the Central Himalaya, and this was attributable to the interaction of geophysical and eco‐evolutionary processes (Qian et al., 2019); the patterns observed at low, middle, and high elevations were hypothesized to be a result of plate tectonics, niche conservatism, and trait convergence, respectively. Based on a large panel of native angiosperm species in the Himalaya, Rana, Price, et al. (2019) observed that phylogenetic clustering increased with increasing elevation, a pattern that was recently supported by studies conducted along other elevational gradients (Qian et al., 2021; Yue & Li, 2021). Despite the clarity in some of these patterns of phylogenetic diversity, the precise evolutionary mechanisms driving elevational diversity remain unknown.

## 3 IMPACT OF CLIMATE CHANGE ON PLANT DISTRIBUTION

### 3.1 Range dynamics under past climate change

The Asian climate is greatly influenced by the extent and height of the Himalaya and the Tibetan Plateau (e.g., Boos & Kuang, 2010; Farnsworth et al., 2019; Molnar et al., 2010). Aeolian and marine sediments from China, India, and the Northern Pacific have revealed three distinct sequential stages of the evolution of Asian climate as a consequence of the Himalaya uplift: enhanced aridity in the Asian interior and associated changes in Asian monsoon dynamics (*ca*. 23 Ma) (Figure 1c) (including the intensification of the East Asian summer and winter monsoons), increased dust transport to the North Pacific Ocean (*ca*. 3.6–2.6 Ma), and, finally, increased variability and weakening of the Indian and East Asian summer monsoons (since 2.6 Ma) (An et al., 2001). In addition, a recent study demonstrated that growth of north and northeastern Tibet may have altered the monsoon system (Li et al., 2021).

Although past climates have been difficult to reconstruct, several hindcasts based on modern pollen assemblages do exist. For instance, Ghosh et al. (2017) used surface samples from an eastern Himalayan elevation gradient for quantitative paleoclimate reconstruction and showed that temperature was the dominant controlling factor for changes during the Last Glacial Maximum. A similar reconstruction based on fossil leaves suggested the existence of a tropical evergreen forest and a warm humid climate at low elevations in the Eastern Himalaya during the Plio‐Pleistocene (Khan et al., 2011). Other recent leaf fossil‐based studies in the eastern Himalaya (Bhatia et al., 2021; Hazra et al., 2020) reported a slightly warmer, wetter, and more humid Neogene climate in the region, although Khan et al. (2019) found that overall, the regional climate has been remained unchanged over the last 15 million years. In terms of glaciation history, it is a matter of consensus that East Asia was generally less glaciated compared to North America and Europe (Shi & Yao, 2002), with most of the glaciers being localized in the Himalaya and the Tibetan Plateau. However, the precise aspects of the extent and timing of the Quaternary glaciations on the Himalayan–Tibetan orogen are still a matter of debate (Muellner‐Riehl, 2019; Owen et al., 2008; Owen & Dortch, 2014; Renner, 2016).

Pleistocene glaciations certainly played a central role in the evolution and divergence of Himalayan species, generally via range contraction (e.g., Liu et al., 2017; Meng et al., 2008; Wang et al., 2016). However, Pleistocene glacial maxima are also believed to have caused range expansions, especially in cold‐tolerant species. Such range expansions in turn may have led to morphological and genetic differentiation driven by topographic and climatic isolation during the subsequent warmer periods (Hewitt, 2004), a mechanism that has been convincingly demonstrated in the Himalaya for cold‐tolerant *Abies* (Peng et al., 2015) and *Taxus* (Liu et al., 2013). Additionally, genomic phylogeography and modeling of *Primula tibetica* identified four distinct lineages, each of which had experienced bottlenecks and multiple expansions during the Pleistocene (Ren et al., 2017). These accounts strengthen the thinking that the “species pump” effect (cycles of expansion and contraction that drive species radiations), driven by orbital variations, could be one of the key drivers of montane biodiversity (Muellner‐Riehl, 2019; Spicer, Farnsworth, et al., 2020).

Generally, the Eastern Himalaya and the Hengduan Mountains provided refugia for many plant species during the Quaternary glaciations (Liu et al., 2012; Qiu et al., 2011) although some other studies have argued in favor of persistence of in situ refugia on the plateau platform (e.g., Opgenoorth et al., 2010; Shimono et al., 2010; Wang et al., 2009), from which species later expanded, possibly to the Himalaya. The distribution range of *Sinopodophyllum hexandrum* became fragmented into in situ refugia in the Eastern Himalaya and Hengduan Mountains during the glacial periods and has not merged again due to topographic isolation imposed by the Mekong‐Salween Divide in the Hengduan Mountains (Li et al., 2011). Meng et al. (2015) found that Pleistocene climatic changes and mountain glaciers, rather than topographic isolation, played the primary role in shaping the spatial distribution of *Oxyria sinensis* in the Himalaya–Hengduan Mountains.

Though the general pattern of species response to paleoclimatic changes in the Himalaya region was “glacial contraction/post‐glacial expansion,” some species have shown unusual demographic histories: For instance, Fu et al. (2020) demonstrated an anomalous range expansion for *Gentiana veitchiorum* and *G*. *lawrencei* var. *farreri* from Last Interglacial (LIG; 127–116 Ka) to present period. Moreover, the potential ranges of some species show the contrary pattern, expanding during glacial maxima and contracting during interglacials (e.g., *Taxus wallichiana*; Liu et al., 2013). Glacial cycles, however, did not alter the distribution ranges of all species; for example, Luo et al. (2016) found that broad‐scale distributions of four subnival perennial herb species generally remained stable over the last glacial/interglacial cycle.

This mosaic of responses by plant taxa to paleoclimatic changes could have important lessons for future phylo‐ and biogeographic studies in the Himalaya, and perhaps other regions. However, caution should be observed when generalizing about the effect of past climate changes based on single‐species data; it is obvious that different species would have varying adaptive potential, hence the variance in response dynamics, as recently demonstrated for Central African rain forest plants (Helmstetter et al., 2020). Therefore, modeling for responses to future climate change should anticipate these differences among taxa, formulating appropriate hypotheses and designing the relevant experiments to test these.

### 3.2 Future changes in species distributions due to climate change

Predicting suitable climatic niches of species and exploring their range dynamics under future climate scenarios is critical for assessing impacts of climate change and hence in prioritizing areas for conservation. For the Himalaya, several studies have shown that future climate changes will alter significantly the distributions of plant species in the region and disrupt biotic interactions (Bobrowski et al., 2017; Chakraborty et al., 2016; Rana et al., 2020; Ranjitkar et al., 2014). For instance, the upper subalpine belt in the Western and Central Himalaya regions has been identified as providing suitable areas for *Betula utilis*, with potential range losses predicted on the eastern side of Himalaya due to competition from *Rhododendron* (Bobrowski et al., 2017). Conversely, *Lobaria pindarensis* is predicted to expand to the northeast, but lose 30%–70% of its current habitat in future (Devkota et al., 2019). He et al. (2019) predicted a northward and westward range expansion for *Meconopsis* by 2070, although the most rapid possible warming scenario showed either range contraction or insignificant change in distribution. For the endangered *Taxus wallichiana*, suitable habitats in the Central Himalaya will rapidly contract (Poudel et al., 2014).

Contrary to the above examples, a study on four forest tree species (*Quercus leucotrichophora*, *Q*. *semecarpifolia*, *Q*. *floribunda*, and *Pinus roxburghii*) did not show any obvious synchronicity in their predicted future response to climatic changes, with species showing both increases and decreases in suitable habitat range (Chakraborty et al., 2016). Furthermore, the distribution ranges of some Himalayan species (e.g., *Q*. *semecarpifolia*) are predicted to remain unchanged under future climate scenarios (Chitale & Behera, 2019). Predictions for the Himalaya have shown that the potential distributions and ecological niches of invasive alien plant species will generally be conserved and, in some cases, expand (Thapa et al., 2018). Although range expansions will generally promote species resilience (Corlett and Westcott, 2013), range expansion of invasive species poses potential ecological threats to native ecosystems.

Because all of the mentioned studies show contradicting trends, it is difficult to obtain a holistic picture of the predicted range shifts of Himalayan species. As alluded to earlier (in Section 3.1), this inconsistency warrants further robust research in the Himalaya, with special consideration to differences in species attributes such as elevational distribution range, heat tolerance, pollination modes, dispersal abilities, and other adaptive traits (Helmstetter et al., 2020). Nevertheless, it is clear from these examples that future climate change will certainly lead to spatial changes in species distributions in the Himalaya and that conservation programs should therefore take these anticipated shifts into consideration.

Another important biogeographic aspect for montane flora is elevational range shifts in which the majority of species are driven to higher elevations as the climate warms (Pauli et al., 2012). This happens because temperatures drop with rising altitude, thus permitting the local survival of cold‐adapted species at relatively higher altitudes during warm epochs. Telwala et al. (2013) provided the first ever evidence of recent warmer winters in the Eastern Himalaya based on a two‐century dataset and reported increased species richness in the upper alpine zone since the 19th century, with a mean upward displacement rate of 28 ± 22 m/decade. Recently, He et al. (2019) predicted a higher rate for *Meconopsis* (21–93 m/decade).

These anticipated upward shifts by montane plant species could be associated with range expansions for the majority of the species involved: an observation that represents a paradigm shift from the traditional view of smaller range sizes at higher altitudes (Liang et al., 2018). This is because the Himalaya exhibits an hourglass type of mountain hypsographic morphology, that is, with more area at high and low than middle altitudes, meaning there would be more available space for upward‐bound species (Elsen & Tingley, 2015; Muellner‐Riehl, 2019). However, continued and unchecked global warming will present an imminent extinction risk as the scope for upward movement will be finite, especially since most of the available space for range expansion in the Himalayan falls behind the mountain range's rain shadow (Figure 2a). Furthermore, the shifting species may encounter new biotic and abiotic pressures to which they could be maladapted, thus disrupting ecosystem balance and stability (Harley, 2011).

Rising temperatures and melting of glaciers in Himalaya have occasioned boundary shifts for high‐elevation ecosystems (Xu et al., 2009). Treeline environments in the Himalaya are highly dynamic, posing difficulties for generalizations on local treeline sensitivity and response to climate change (Schickhoff et al., 2015). Nevertheless, these treelines have been used widely in the prediction and reconstruction of climate patterns in the Himalaya (e.g., Dawadi et al., 2013; Gaire et al., 2017; Hamid et al., 2018; Yadav & Singh, 2017). Most of these studies were conducted in the Western Himalaya and along the southern slopes, meaning that the available literature lacks contributions from the Eastern Himalaya.

## 4 CONSERVATION AND MANAGEMENT

In light of the link between biodiversity, ecosystem health, and human well‐being (Faith et al., 2010; Molina‐Venegas et al., 2021), conservation interventions in species‐rich ecosystems such as the Himalaya should be strengthened. Satyal et al. (2017) painted a grim picture of the current status of Himalayan biodiversity conservation plans, arguing that these employ an overly academic and technical approach to biodiversity protection. Hence, Satyal et al. (2017) proposed the adoption of multifaceted, integrative, and policy‐backed approaches. The fact that the Himalaya spans several nations presents an obstacle to conservation efforts and to the holistic understanding of the biogeography of Himalayan flora, largely because researchers are often limited to gathering data and material within only one country. This challenge is exacerbated by political instability and territorial disputes among countries across the Himalaya. Therefore, transboundary research initiatives as well as biodiversity monitoring networks should be conceived urgently and implemented quickly in order to circumvent these challenges (Liu, Milne, Cadotte, et al., 2018). Moreover, an integrated multidisciplinary approach to biodiversity conservation should be adopted; for instance, through the establishment of science–policy partnerships or even the signing of intergovernmental biodiversity treaties (Marchese, 2015).

As part of immediate conservation measures, we propose that more effort should be made toward the protection of elevational gradients in the Himalaya, particularly in light of the recent finding that Asian mountain ranges currently have a relatively low proportion of altitudinal ranges under protection (Elsen et al., 2018). In situ conservation of species should be directed more at elevations of about 2,000–4,000 m, where most of the species diversity occurs, while at the same time limiting ecologically unsustainable anthropogenic activities in the lowlands. For long‐term planning, however, high‐elevation belts should also be protected, especially above areas that are currently protected, as many species are predicted to shift upward with a warming climate. Moreover, biodiversity conservation efforts are often curtailed by unchecked anthropogenic activities, especially at lower elevations. These lower mountain slopes may act as future elevational refugia for species adapted to warm climates, so additional protected areas should be set up at these low altitudes in order to mitigate the effects of human activities. Furthermore, stock‐taking of resident taxa coupled with policy‐guided protection of species should be incorporated in the conservation strategy. Despite some efforts toward taking biodiversity inventories of plant species in the Himalaya (Khuroo et al., 2010), still much remains to be done with regard to identification and protection of endangered taxa, especially those of economic value such as medicinal plants (Tali et al., 2018). Establishment of standard DNA barcode reference libraries will augment the efforts of law enforcers in the protection of such species in the Himalaya (e.g., Liu, Milne, Moller, et al., 2018) and possibly reveal cryptic diversity.

Future persistence of some species will be dependent upon their respective abilities to colonize new habitats, mostly via natural dispersal corridors and/or along ecological gradients. However, owing to increased fragmentation of habitats, these natural dispersal routes may be interrupted, impeding movement and increasing the risk of extinction for many species in fragile ecosystems such as the Himalaya. To avert this threat, conservationists should scale up efforts toward ecosystem conservation, which is a holistic approach that goes beyond the intrinsic properties of individual species and ensures preservation of the complex web of biotic codependencies that are critical for healthy ecosystem function (Spicer, Farnsworth, et al., 2020). However, for plant species with relatively poor dispersal abilities, we propose the adoption of artificial dispersal strategies (e.g., Liden et al., 2004; Wallin et al., 2009; but see Gaitán‐Espitia & Hobday, 2021). On a related note, invasive and alien plant species pose a significant ecological risk in the Himalaya and therefore also requires urgent intervention. Furthermore, we recommend that future studies explore the mechanisms underlying narrow endemism of plant species in the Himalaya, with a particular focus on how such species have responded to past climate change and hence might do so in the future.

## 5 PROSPECTS AND OUTSTANDING QUESTIONS

Addressing the uncertainty surrounding the number of species in the Himalaya should be a key priority area for future research in the region, owing to the unending controversy surrounding this topic. One likely cause for this variation between estimates is that different studies delimit the region in different ways. More accurate estimates of species numbers could be achieved in future by carrying out adequate sampling across the entire Himalaya range while adopting a common and standard boundary for the Himalaya to ensure uniformity and guarantee reproducibility in species estimation studies. As a caveat to this point, however, the description of new species should be done using an integrative taxonomic approach (Dayrat, 2005), that harnesses the power of multiple lines of evidence such as morphological, molecular, and ecological data. Such a unified approach will guard against the current avalanche of biologically meaningless and taxonomically unsupportable supposed species being newly described in biodiversity hotspots, most of which end up diverting resources that would otherwise have been used to conserve the truly deserving taxa. To further strengthen conservation programs, it would be important to establish species checklists and their respective DNA barcode libraries that exclude spurious taxa, but include associated distribution information for well‐circumscribed species. Such data will in turn be useful in guiding conservation prioritization in the Himalaya.

Despite the clear role of ecological barriers, the exact details of the underlying isolation mechanisms, and precise evolutionary consequences thereof, remain to be worked out in the region. For instance, the relative contributions of climatic and geographic barriers need to be clearly defined in the Himalaya. Moreover, more investigations should explore the role of the east‐west orientation of the Himalaya, as this will complement the current knowledge on past and potential species range shifts, thus enabling a broader understanding and providing sharper insights into the effect of vicariance factors on the biogeographical, speciation and extinction dynamics in the Himalaya.

To clarify these patterns, future investigations should employ landscape genomics across spatial and temporal scales, as recently reviewed by Fenderson et al. (2019); for instance, genomic analysis of current and historical samples may help to untangle the determinants of genetic structure, hence shed more light on the evolutionary mechanisms of plants in the region. On a related note, it would be desirable to design more empirical biogeographical studies based on species that are exclusively endemic to the Himalaya, in order to specifically test for the presence and role of topographic and climatic barriers to intraspecific gene flow.

Biogeographical investigations in Himalaya are characterized by two major drawbacks: 1) utilization of single species with unidimensional approaches and 2) a geographical bias favoring the Indian Himalaya. To resolve these challenges, future studies should utilize multiple taxa collected from the entire Himalaya region in order to provide deeper insights into the biotic assembly and evolution of Himalayan flora. Moreover, investigators need to adopt an integrative approach in which multifaceted methods are applied at higher organizational levels such as communities or even entire ecosystems as recommended previously by Favre et al. (2015). Integrative approaches encompassing tools such as genomics, species distribution modeling, paleobotany, geology, climatology, and sociology can offer more meaningful insights into the evolution and diversification of the Himalayan flora, as well as its response to past and future climate change.

Many of the reviewed studies on species richness along elevational gradients were based on pre‐recorded species occurrences via specimens in herbaria and virtual databases or monograph records. These approaches are not only retrospective, but also prone to sampling bias due to the lack a standard sampling protocol. In light of this, we propose the adoption of methods that utilize current primary data from actual field observations linked to permanent elevational gradient plots (e.g., Luo et al., 2019). Furthermore, the role of altitudinal gradients in driving speciation is yet to be fully explored in the Himalaya. In particular, uncertainty remains about the link between speciation rate and the midelevation peaks of species richness, and how the pattern of beta diversity (species turnover) is driven by factors related to elevation; these should therefore be mainstreamed in future research plans for the region.

In view of the importance of anthropogenically driven disturbances in the Himalaya region, more research effort should be directed toward disentangling the various human activities and their relative roles in disrupting ecosystem services in the region. In this regard, it would be useful to design sociological studies and develop tools that directly engage various local stakeholder groups such as farmers, foresters, the tourism industry, and policy makers (e.g., Heath et al., 2020; Paudel et al., 2020). The assessment of the role of climate change in shaping the spatiotemporal patterns of species diversity, phylogenetic diversity, and genetic diversity is a research frontier that remains unexplored in the flora of the Himalaya, or even the East Asian mountains, despite the huge attention the topic has received in Europe and North America. For this particular purpose, the Himalaya presents an ideal opportunity due to its relatively high species richness coupled with its high sensitivity to environmental changes. Moreover, with the increasing frequency of wildfires in the Himalaya and its surroundings (You et al., 2018), modeling the effect of wildfires on Himalayan taxa, particularly in light of the likely changes in the Indian summer monsoon and the Westerlies, could be an important research direction in the near future. Murthy et al. (2019) made a first attempt at this in the Himalayan lowlands and hence provided a framework for similar investigations in the region.

## CONFLICT OF INTEREST

None declared.

## AUTHOR CONTRIBUTION

**Moses C. Wambulwa:** Conceptualization (equal); Writing‐original draft (lead). **Richard I Milne:** Writing‐original draft (supporting); Writing‐review & editing (equal). **Zeng‐Yuan Wu:** Conceptualization (equal); Writing‐review & editing (equal). **Robert A. Spicer:** Writing‐original draft (supporting); Writing‐review & editing (equal). **Jim Provan:** Writing‐original draft (supporting); Writing‐review & editing (equal). **ya huang luo:** Conceptualization (equal); Writing‐original draft (supporting). **Guang‐Fu Zhu:** Conceptualization (equal); Software (lead); Writing‐review & editing (equal). **Wan‐Ting Wang:** Software (equal); Writing‐review & editing (equal). **Hong Wang:** Funding acquisition (equal); Supervision (equal); Writing‐review & editing (equal). **Lian‐Ming Gao:** Funding acquisition (supporting); Writing‐review & editing (equal). **De‐Zhu Li:** Conceptualization (equal); Funding acquisition (supporting); Supervision (lead); Writing‐review & editing (equal). **Jie Liu:** Conceptualization (equal); Funding acquisition (lead); Writing‐original draft (supporting).

## Data Availability

Not applicable.
